# Number of contiguous vertebral cross-links in the spine indicates bone formation: a cross-sectional study

**DOI:** 10.1186/s12891-023-06833-9

**Published:** 2023-09-15

**Authors:** Mitsuru Furukawa, Reo Shibata, Kunimasa Okuyama

**Affiliations:** 1 Department of Orthopedic Surgery, Murayama Medical Center, Tokyo, Japan; 2 Department of Orthopedic Surgery, Shizuoka City Shimizu Hospital, Shizuoka, Japan

**Keywords:** Diffuse idiopathic skeletal hyperostosis, Femur proximal bone mineral density, Type I procollagen N-terminal propeptide, Bone metabolic markers, Tartrate-resistant acid phosphatase 5b

## Abstract

**Background:**

As an indicator to evaluate the risk of fracture in diffuse idiopathic skeletal hyperostosis, the maximum number of vertebral bodies’ bone cross-linked with contiguous adjacent vertebrae (max VB) was developed. This study retrospectively investigates the relationship between max VB, bone mineral density (BMD), and bone metabolic markers (BMM).

**Methods:**

In this cross-sectional study (from April 2010 to January 2022), males (*n* = 114) with various max VB from the thoracic vertebra to the sacrum, measured using computed tomography scans, were selected to assess femur BMD and BMM. The association of max VB with the total type I procollagen N-terminal propeptide (P1NP), tartrate-resistant acid phosphatase 5b (TRACP-5b), and bone turnover ratio (BTR = TRACP-5b/P1NP) as well as its relationship with femur BMD with P1NP and TRACP-5b, were investigated. Furthermore, the relationship between P1NP and TRACP-5b was investigated.

**Results:**

P1NP increased in proportion to max VB and TRACP-5b increased in proportion to P1NP. Moreover, BTR was inversely proportional to max VB. Finally, femur BMD was inversely proportional to P1NP and TRACP-5b.

**Conclusion:**

As max VB increased with P1NP—a potential osteogenesis indicator—and BTR was inversely proportional to max VB with compensatory TRACP-5b increase, max VB can be considered as a possible predictor of bone fusion.

## Introduction

In diffuse idiopathic skeletal hyperostosis (DISH), a continuous and measurable osteogenesis process involves development from incomplete and pointy bone bridges to more flowing and complete bridges [1]. The formation of bone cross-links is caused by ectopic ossification in which the anterolateral ligament component of the vertebral bodies becomes an osteophyte [2]. Diseases with high propensity for ossification, such as ossification of posterior longitudinal ligament, are reported to have high bone density, but total type I procollagen N-terminal propeptide (P1NP) and tartrate-resistant acid phosphatase 5b (TRACP-5b) are reported to be lower than controls and have low metabolic turnover [3–6]. Alternatively, there are reports of bone mineral density (BMD) and bone metabolism markers (BMM) related to DISH, but no common view has been reached. Some previous reports have shown that BMD is unchanged or higher in patients with DISH than in controls, i.e., people without DISH [7–12]. With respect to bone BMM, some studies have reported higher intact parathyroid hormone levels but lower P1NP and serum sclerostin levels, while others have reported lower dickkof-1 levels, which suppress sclerostin; however, the results in none of these reports were conclusive [3, 13, 14]. The reason for such disparate results is that although DISH, which is based on whether a patient presents with or without spinal disease with a bone bridge that fits Resnick’s definition [15], involves several different populations that were analyzed together [16]. To elucidate the mechanism of DISH, we devised the maximum number of vertebral bodies involved in bone cross-linked with contiguous adjacent vertebrae without any interruption (max VB) [17]. Using the max VB index, the degree of bone cross-linking can be evaluated in 18 steps. This study aimed to investigate the impact of max VB on BMD and BMM.

## Materials and methods

### Study design

This retrospective cross-sectional study was conducted in Japan.

### Study participants

Between April 2010 and January 2022, a total of 20,357 patients visited the Department of Orthopedic Surgery, Shizuoka City Shimizu Hospital (Shizuoka, Japan). Of these, 2176 were tested for BMD by dual-energy X-ray absorptiometry (GE Medical Systems Lunar, Chicago, IL, US). Overall, BMM was evaluated in 312 male patients. Only male patients were selected to eliminate the effects of postmenopausal osteoporosis because DISH occurs more frequently in men. Computed tomography (CT) scans (Discovery CT 750HD, GE Healthcare) of the thoracic to lumbar vertebrae and pelvis were obtained from 179 patients. By examining medical records, examination interviews, and phone interviews, we identified 132 patients without an exclusion criterion, i.e., history of hyperparathyroidism and rheumatism, steroid use, and osteoporosis drug treatment. Of 132 cases, we excluded 14 patients with sacroiliac (SI) joint ankylosis, leaving 118 patients without ankylosis. Of these 118 patients, 114 (mean age, 77 ± 9.1 years; range, 50–98 years) were selected from among male patients only, excluding 4 who had fractures within 1 year of the BMM collection.

### Study variables

For the 114 patients, CT scans were obtained from the thoracic vertebra to the pelvis. CT is mainly taken for close examination of lumbar back pain and for determining surgical procedures for degenerative diseases of the spine. In addition, max VB was evaluated from the thoracic vertebra to the sacrum in consultation with three orthopedic surgeons. As per Resnick’s definition, the DISH group included patients with four or more consecutive vertebral cross-links and the non-DISH group included those with fewer than four consecutive vertebral cross-links [15]. Besides, age and bone mass index (BMI) were obtained from the medical questionnaire filled out at the time of the initial visit. The following items were selected based on the blood data collected: total serum calcium concentration, serum phosphorus concentration, TRACP-5b, P1NP, estimated glomerular filtration rate (eGFR), hemoglobin A1c (HbA1c), and femur BMD was estimated by the total. The bone turnover ratio (BTR) is defined in previous papers as TRACP-5b divided by P1NP [18].

### Contents for investigation

First of all, we investigated the timing of CT, bone mineral density, and bone metabolism markers to investigate the interval between each. Next, the mean and standard deviation were compared with the following items: age, BMI, total serum calcium concentration, serum phosphorus concentration, TRACP-5b, P1NP, eGFR, HbA1c, femur BMD, and BTR between the DISH (*n* = 69) and non-DISH (*n* = 45) groups without adjustment. Furthermore, the outcomes influenced by max VB (*n* = 114) were investigated. Notably, the outcomes examined were P1NP, TRACP-5b, and BTR, and the confounding factors were age, eGFR, and HbA1c. Furthermore, we examined whether exposure to P1NP and TRACP-5b influenced femur BMD, with age, eGFR, HbA1c, and BMI as the confounding factors. Finally, the influence of P1NP on TRACP-5b was examined with age, eGFR, and HbA1c as confounders.

### Statistical analysis

In this study, statistical analyses were performed using SPSS ver. 26 (IBM Corp., Armonk, NY) and statistical software R-4.0.3 (Index of /src/base/R-4 [r-project.org]). Missing data are 1; eGFR, 4; HbA1c. In addition, comparisons between the DISH and non-DISH groups were conducted for each item using the Mann–Whitney *U* test. Moreover, the outcomes significantly affected by exposure were tested using multiple linear regression. Notably, *P* < 0.05 was considered statistically significant in all analyses.

## Results

The mean and standard deviation of the timing of CT and BMD were 57 ± 82 days (min 0 and max 364), CT and BMM were 49 ± 79 days (min 0 and max 338), and BMD and BMM were 23 ± 49 days (min 0 and max 287). Overall, the subjects’ mean age was 77 ± 9 years, P1NP 53 ± 28 ng/ml, TRACP-5b 433 ± 177 mU/dl, BTR 9 ± 3.5, eGFR 60 ± 18 ml/min/1.73 m^2^, HbA1c 6 ± 0.6%, max VB 5.9 ± 4.7, femur BMD 0.9 ± 0.2 g/cm^2^, and BMI 19.5 ± 3.1 kg/m^2^. The number of max VB was as follows: 0 (*n* = 19), 2 (*n* = 10), 3 (*n* = 16), 4 (*n* = 14), 5 (*n* = 3), 6 (*n* = 8), 7 (*n* = 6), 8 (*n* = 6), 9 (*n* = 5), 10 (*n* = 2), 11 (*n* = 4), 12 (*n* = 8), 13 (*n* = 5), 14 (*n* = 4), 15 (*n* = 2), and 17 (*n* = 2). The number of cases per max VB was evenly divided. Age, eGFR, max VB, P1NP, femur BMD and BTR remarkably differed between the DISH and non-DISH groups in an unadjusted comparison (Table 1).

**Table 1:** Comparison of each item in DISH and non-DISH

	Non-DISH	DISH	*P*
Number	45	69	
Age	74.6 ± 7.5	78.6 ± 9.7	< 0.05*
BMI (kg/m^2^)	19.1 ± 2.9	19.8 ± 3.2	n.s.
max VB	1.5 ± 1.3	8.9 ± 3.7	< 0.01**
Femur BMD (g/cm^2^)	0.91 ± 0.15	0.96 ± 0.21	< 0.05*
P1NP (ng/ml)	44.1 ± 17.8	58.7 ± 31.9	< 0.01**
TRACP-5b (mU/dl)	433 ± 182.9	432.4 ± 171	n.s.
Ca (mg/dl)	9.1 ± 0.4	9 ± 0.4	n.s.
P (mg/dl)	3.1 ± 0.6	3.1 ± 0.5	n.s.
eGFR (ml/min/1.73m^2^)	63.8 ± 14.3	58.2 ± 19.6	< 0.01**
HbA1c (%)	6 ± 0.6	6 ± 0.7	n.s.
BTR (mU•ng/dl•ml)	10.4 ± 4	8.1 ± 2.8	< 0.01**

*P < 0.05, **P < 0.01 was significant

BMD, bone mineral density; BMI, bone mass index; BTR, bone turnover ratio; DISH, diffuse idiopathic skeletal hyperostosis; eGFR, estimated glomerular filtration rate; HbA1c, hemoglobin A1c; max VB, the maximum number of vertebral bodies’ bone cross-linked with contiguous adjacent vertebrae; P1NP, total type I procollagen N-terminal propeptide, TRACP-5b, tartrate-resistant acid phosphatase 5b

**Fig. 1:**
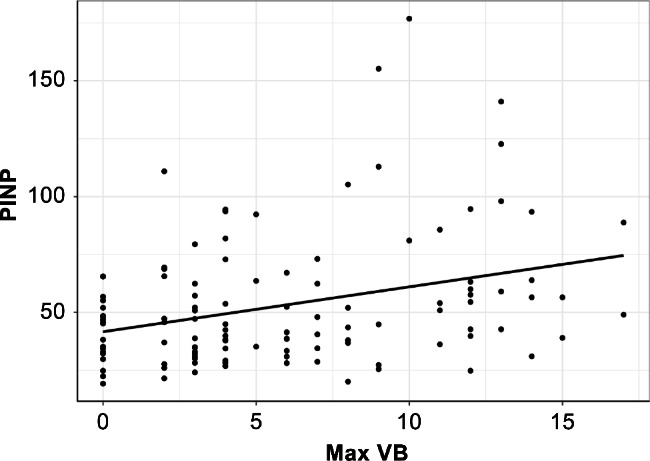
Scatterplots of max VB versus P1NP. P1NP increased in proportion to max VB. max VB, the maximum number of vertebral bodies’ bone cross-linked with contiguous adjacent vertebrae; P1NP, total type I procollagen N-terminal propeptide

The results of this analysis suggested that max VB may have influenced the values of P1NP and femur BMD. Therefore, we first investigated the relationship between max VB and P1NP with age, eGFR, and HbA1c as confounding factors. We found that only P1NP increased in proportion to max VB (*Correlation coefficient* = 0.05, *P* = 0.003; Fig. 1; Table 2).

**Table 2:** Relation between max VB and P1NP

Characteristic	Correlation coefficient	95% CI	*P*
P1NP (ng/ml)	0.05	0.02, 0.08	0.003**
Age	0.02	−0.09, 0.12	0.8
eGFR (ml/min/1.73m^2^)	−0.01	−0.07, 0.04	0.6
HbA1c (%)	−0.83	−2.2, 0.51	0.2

*P < 0.05, **P < 0.01 was significant

eGFR, estimated glomerular filtration rate; HbA1c, hemoglobin A1c; max VB, the maximum number of vertebral bodies’ bone cross-linked with contiguous adjacent vertebrae; P1NP, total type I procollagen N-terminal propeptide

**Fig. 2:**
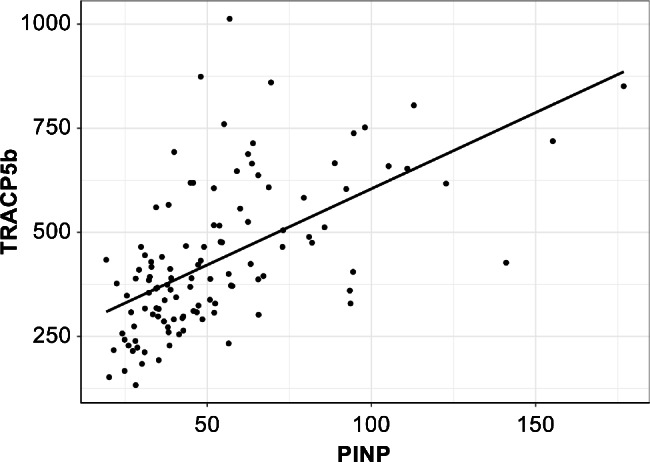
Scatterplots of P1NP versus TRACP-5b. TRACP-5b increased in proportion to P1NP. P1NP positively correlates with TRACP-5b. P1NP, total type I procollagen N-terminal propeptide; TRACP-5b, tartrate-resistant acid phosphatase 5b

**Fig. 3:**
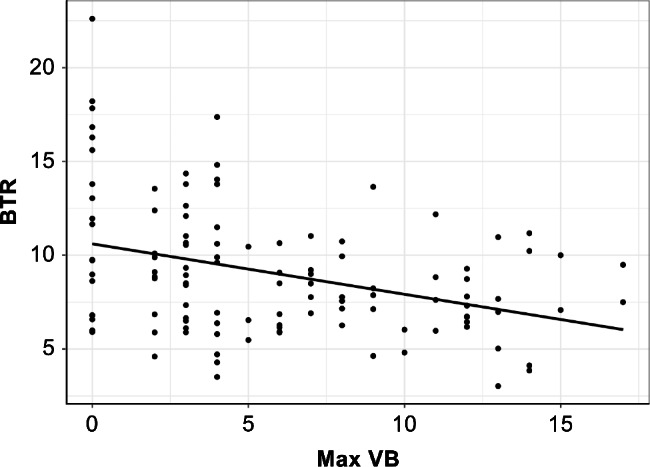
Scatterplots of max VB versus BTR. BTR (TRACP-5b/P1NP) was inversely proportional to max VB. BTR, bone turnover ratio; max VB, the maximum number of vertebral bodies’ bone cross-linked with contiguous adjacent vertebrae; P1NP, total type I procollagen N-terminal propeptide; TRACP-5b, tartrate-resistant acid phosphatase 5b

Besides, no significant correlation was obtained between max VB and TRACP-5b, whereas TRACP-5b increased in proportion to P1NP (*Correlation coefficient* = 0.1, *P* < 0.001; Fig. 2). BTR was inversely proportional to max VB (*Correlation coefficient* = − 0.48, *P* < 0.001; Fig. 3). Moreover, femur BMD was inversely proportional to P1NP and TRACP-5b (P1NP: *Correlation coefficient* = − 0.14, *P* = 0.018; TRACP-5b: *Correlation coefficient* = − 0.03, *P* = 0.002; Table 3).

**Table 3:** Relation with femur BMD and bone metabolic marker

Characteristic	Correlation coefficient	95% CI	*P*
**femur BMD vs. P1NP**			
P1NP (ng/ml)	−0.14	−0.26, − 0.02	0.018**
Age	−0.49	−0.92, − 0.06	0.026*
eGFR (ml/min/1.73cm^2^)	−0.31	−0.51, − 0.11	0.002**
HbA1c (%)	3.0	−2.1, 8.1	0.2
BMI (kg/m^2^)	1.7	0.58, 2.8	0.003**
**Femur BMD vs. TRACP-5b**			
TRACP-5b (mU/dl)	−0.03	−0.05, − 0.01	0.002**
Age	−0.43	−0.85, 0.00	0.051
eGFR (ml/min/1.73m^2^)	−0.32	−0.51, − 0.12	0.002**
HbA1c (%)	2.7	−2.3, 7.6	0.3
BMI (kg/m^2^)	1.3	0.21, 2.4	0.02*

*P < 0.05, **P < 0.01 was significant

BMD, bone mineral density; BMI, bone mass index; eGFR, estimated glomerular filtration rate; HbA1c, hemoglobin A1c; max VB, the maximum number of vertebral bodies’ bone cross-linked with contiguous adjacent vertebrae; P1NP, total type I procollagen N-terminal propeptide; TRACP-5b, tartrate-resistant acid phosphatase 5b

## Discussion

### Factors influencing maxVB, BMM and BMD and the validity of this study

The incidence of DISH increases with age and the number of bone cross-links [1]. Although BMM, BMD, and CT from the thoracic to the lumbar spine were not necessarily taken at the same time, the average interval between inspection dates was 1–2 months, and all inspections were performed within a year. The number of bone bridges reported to develop over the mean course of the five-year period suggests that a gap of approximately one year is not so significant [1]. In addition, studies have reported that age, renal function, postmenopausal osteoporosis, history of hyperparathyroidism, rheumatism, and history of steroid and osteoporosis medication are factors affecting P1NP and TRACP-5b [19, 20]. Thus, the eligible patients were eliminated according to the patient’s medical records or subsequent telephonic interviews. Since we are dealing only with male patients and do not need to consider postmenopausal osteoporosis, a rapid decline in bone mineral density is unlikely to occur within a year or so. Furthermore, since no patients were treated for osteoporosis, the effect of bone metabolism markers was considered small. Finally, in a previous study, BMM did not increase until the first week following a fracture but remained elevated up to 1 year following the fracture [21, 22]. Hence, patients with fractures occurring within one year were also excluded from this study, suggesting that we eliminated as many factors as possible that could affect BMM.

### Differences in BMM between DISH and ankylosing spondylitis (AS)

AS and DISH involve bone cross-linking between the vertebrae, but show completely different levels of BMD and BMM. Among BMM, bone resorption markers, such as serum C-telopeptides, which belong to type I collagen (CTX), are high in AS. Of note, higher CTX signifies lower bone density values. Both AS and DISH are similar in terms of the formation of bone bridges in the spine when bone resorption markers increase, but they differ in bone density and P1NP [23–25]. The elevated bone resorption markers in both diseases are different, and the augmented bone resorption in AS induces the decreased bone density, whereas bone resorption in DISH increases at the expense of accelerated bone formation. First, it is essential to distinguish AS as a prerequisite for assessing bone healing in DISH; for this, the modified New York criteria evaluated by clinical and imaging items and human leukocyte antigen B27 (HLA-B27) have been used previously [26, 27]. In this study, all tests were conducted within insurance, but HLA-B27 was not tested. Nevertheless, as the incidence of DISH is very high compared with AS, which is remarkably low (0.48/100,000), in the Japanese population, and because patients without SI joint fusion were examined in this study, the population is considered AS-free [28]. The results of this study further showed that bone density was higher in the DISH group and that only P1NP increased in proportion to max VB, with TRACP-5b showing a compensatory rise. These increases were also considered to represent DISH as they were different from the trends of BMD and BMM in AS.

### Effect of max VB on P1NP

To the best of our knowledge, no paper has yet examined the effect of the number of bone cross-links on BMM. Moreover, as mentioned above, studies about BMM comparing DISH and non-DISH have not eliminated confounding factors [3, 13, 14]. P1NP, which is produced from the early stage of osteoblast differentiation, acutely reflects early bone formation; high P1NP indicates high osteogenic potential, which may be due to ectopic ossification and bone bridging after migration of osteoblast progenitor cells [29]. First, age must be adjusted because it affects both the number of bone cross-links and P1NP. As age increases, the number of bone cross-links increases, whereas P1NP decreases [1, 30]. Given these findings, we would expect P1NP to decrease with increasing max VB. Unexpectedly, P1NP was positively correlated with max VB after adjustment for other confounders. Cases exist where BMM cannot be accurately determined. In cases of spinal fractures, metastatic spinal tumors, diseases involving paralysis of the lower extremities, or bladder rectal disorders requiring urgent surgery, which must be performed without waiting for the BMM results. Moreover, BMM are difficult to determine in certain cases because they fluctuate with age, gender, fractures, autoimmune diseases, and administration of osteoporosis medications [19–21]. Therefore, max VB can be considered an indicator of bone formation and it is particularly useful as an indicator of bone formation when bone metabolic markers are not available or helpful.

### Effect of BMM on BMD

In the present study, BMD showed a negative correlation with both bone formation and resorption markers, suggesting that high BMD and bone formation markers are incompatible. An interesting paper examined the correlation between P1NP levels and hip bone density in teriparatide-treated patients, reporting that approximately 60% of patients presented with less hip BMD, which was independent of the changes in P1NP levels [31]. This fact may support the result that when both bone formation and resorption markers as well as bone metabolic turnover are low, bone density is high. Conversely, when BMM are elevated, bone metabolic turnover is increased and bone density is low. Subsequently, we considered max VB and BMD. Regarding bone density in DISH, there have been reports of higher or unchanged bone density compared with controls [7–12]; however, bone density is higher for max VB from 4 to 8 and unchanged for max VB from 9 to 18 compared with the non-DISH group. Therefore, bone density results in cases of DISH will vary depending on the max VB [16]. This is because when max VB is between 4 and 8, bone formation and compensatory bone resorption are moderate, resulting in high BMD. Conversely, when max VB is between 9 and 18, bone formation and compensatory bone resorption are accelerated, resulting in lower BMD due to increased bone metabolic turnover.

### Can max VB be a predictive indicator of bone healing?

High bone density is reported to be favorable for bone healing [32, 33]. In contrast, inhibitory factors of bone healing are low bone density, underlying factors like steroid use, and smoking history [34, 35]. Aging is a cause of osteoporosis but not necessarily a cause of bone fusion failure in spinal fusion surgery [36, 37]. Reports regarding the effects of drugs on bone fusion have stated that the weekly administration of teriparatide promoted bone union within 6 months following posterior lumbar interbody fusion [38], and there was significantly delayed union after 6 months in long-term bisphosphonate users [39]. While these factors have been reported to influence bone healing, it is not common to predict bone healing by the values of bone metabolic markers. Inoue et al. used BTR (TRACP-5b/P1NP) as an index of bone remodeling and a factor that can predict bone healing in posterior lumbar interbody fusion [18]. Notably, this index was devised because low P1NP and high TRACP-5b levels are risk factors for poor bone remodeling. Although the mechanism of bone healing in posterior lumbar interbody fusion and posterolateral fusion remains unclear, the rate of bone healing is fast-tracked in osteoinductive cages and demineralized bone fibers, which could be considered as ectopic ossification [40–42]. The results of this study, in which BTR was inversely proportional to maxVB, suggested that the increase in maxVB, which may have promoted ectopic ossification, enhanced bone healing.

### Limitations

This study has some limitations that are worth acknowledging. First, this was a retrospective study, with a small sample size. Second, this study was analyzed using P1NP, which reacts at the early stage of bone formation, rather than osteocalcin or bone alkaline phosphatase, which is a marker of the final stage of bone formation, because the study was conducted within insurance. Finally, that bone cross-link formation in DISH is not only affected by factors owing to bone metabolism, but also by degeneration. In the future, it will be necessary to investigate bone healing after surgery for chronic spinal diseases and fractures with the degree of bone cross-linking in a prospective study.

## Conclusions

This study demonstrates that max VB increased in proportion to P1NP, which was considered an indicator of bone formation. Furthermore, there was a compensatory rise in the levels of bone resorption and bone rotation increased, whereas BTR was inversely proportional to max VB, suggesting that it could be a predictor of bone healing. Hence, measuring max VB before the spinal fusion surgery is beneficial to easily confirm the possibility of bone fusion.

## Data Availability

The dataset supporting the conclusions of this article is included within the article.

## Abbreviations

AS: Ankylosing spondylitis
BMD: Bone mineral density
BMI: Bone mass index
BMM: Bone metabolic markers
BTR: Bone turnover ratio
CT: Computed tomography
DISH: Diffuse idiopathic skeletal hyperostosis
PINP: Propeptide of type I procollagen
